# Density and habitat use of one of the last jaguar populations of the Brazilian Atlantic Forest: Is there still hope?

**DOI:** 10.1002/ece3.8487

**Published:** 2022-01-15

**Authors:** Fernando Cesar Cascelli de Azevedo, Juliana Benck Pasa, Ricardo Corassa Arrais, Rodrigo Lima Massara, Cynthia Elisa Widmer

**Affiliations:** ^1^ Departamento de Ciências Naturais Universidade Federal de São João del Rei São João del Rei Brasil; ^2^ Instituto Pró‐Carnívoros Atibaia Brasil; ^3^ Programa de Pós‐Graduação em Ecologia Universidade Federal de São João del Rei São João del Rei Brasil; ^4^ Programa de Pós‐graduação em Ecologia, Conservação e Manejo da Vida Silvestre Departamento de Genética, Ecologia e Evolução Instituto de Ciências Biológicas Universidade Federal de Minas Gerais Belo Horizonte Brasil; ^5^ Projeto Carnívoros do Rio Doce – PCRD Parque Estadual do Rio Doce Marliéria Brasil

**Keywords:** apex predator, biodiversity hotspot, demography, human‐related habitats, landscape variables, population monitoring

## Abstract

The jaguar (*Panthera onca*) plays an important role in maintaining biodiversity and ecological processes. We evaluated the status of a jaguar population in one of the last stronghold habitats for its conservation in the Atlantic Forest, the Rio Doce State Park (RDSP). We used a random survey design from 2016/17 to estimate jaguar abundance and density as well as its occupancy and detection probabilities in the entire Park's area. To monitor for temporal fluctuations in density and abundance, we used a systematic survey design in the southern portion of the Park where jaguars were more recorded when using the random approach. We then conducted two surveys in 2017/18 and 2020. Our 2016/17 random survey revealed that jaguar density (0.11 ± *SE* 0.28 individuals/100 km^2^) was the lowest obtained for the species across the Atlantic Forest. We noticed that jaguar density increased three times from 2017/18 (0.55 ± *SE* 0.45 individuals/100 km^2^) to 2020 (1.61 ± *SE* 0.6 individuals/100 km^2^). Jaguar occupancy and detection probability were 0.40 and 0.08, respectively. The low jaguar occupancy probability was positively associated with smaller distances from lakes and records of potential prey. The detection probability was positively associated with prey detection, the rainy season, and smaller distances from lakes. Our work contributes to a growing awareness of the potential conservation value of a protected area in a human‐dominated landscape as one of the last strongholds for jaguars across the Atlantic Forest.

## Graphical abstract

We evaluated the status of a jaguar population in one of the last stronghold habitats for its conservation in the Atlantic Forest, the Rio Doce State Park (RDSP). Our 2016/17 random survey revealed that jaguar density was the lowest obtained for the species across the Atlantic Forest. The 2017/18 systematic survey revealed density estimates slightly higher. We noticed that jaguar density increased three times from 2017/18 (0.55 ± *SE* 0.45 individuals/100 km^2^) to 2020 (1.61 ± *SE* 0.6 individuals/100 km^2^). Jaguar occupancy and detection probability were 0.40 and 0.08, respectively. Density estimates and occupancy probabilities represent not only a temporal snapshot assessment of jaguar population estimates and habitat use, but rather a first attempt to track population change over time.

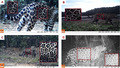

## 1 INTRODUCTION

The jaguar (*Panthera onca*) is the largest apex predator in the Americas and plays an important role in maintaining biodiversity and ecological processes in terrestrial and semiaquatic ecosystems via multiple food pathways (Paviolo et al., 2016; Ripple et al., 2014). As for most large carnivore species, jaguars have the potential role of limiting populations of both medium and large‐sized herbivores through predation and mesocarnivores through intraguild competition (Ripple et al., 2014). Currently distributed from the southern United States to northern Argentina (Paviolo et al., 2016; Sanderson et al., 2002), jaguars are cryptic, solitary, and territorial carnivores that require large home ranges and a stable prey base for their long‐term survival (Morato et al., 2016; Sanderson et al., 2002). Therefore, jaguars occur naturally at low densities (Jędrzejewski et al., 2018). However, habitat loss, depletion of prey base, and human persecution have shrunk jaguars' habitat in more than half of its original occurrence (De la Torre et al., 2017). The species is listed as near threatened in the IUCN Red List (Quigley et al., 2017), but it has already become locally extinct or critically endangered along most of its distribution, particularly in the Brazilian Atlantic Forest, where populations are highly threatened (De la Torre et al., 2017; Galetti et al., 2013; Paviolo et al., 2016).

The Brazilian Atlantic Forest is one of the world's 25 recognized biodiversity hotspots, yet notoriously one of the most devastated, threatened, and understudied ecosystem on the planet (Metzger et al., 2009). Originally covering about 150 million hectares (ha), this biome currently maintains less than 12% of its original forest cover (Ribeiro et al., 2009). Most (~80%) Atlantic Forest remnants are small (<50 ha), isolated and present different stages of forest succession (Metzger et al., 2009; Ribeiro et al., 2009). Based on the species–area relationship (Pimm & Raven, 2000), most of these small fragments are probably depleted of species, particularly for medium‐ and large‐sized mammals such as the jaguar that depend on large areas to survive (Bodmer et al., 1997; Bogoni et al., 2020). Jaguar density estimates have been widely reported at some sites across the Atlantic Forest (Paviolo et al., 2016). This apex carnivore has already been extinct from almost all remaining Atlantic Forest fragments. It is estimated that there are less than 300 jaguars persisting in around only 2.8% of the remaining Atlantic Forest biome (Haag et al., 2010; Paviolo et al., 2016).

One of the last major remnants of Atlantic Forest in Brazil is the Rio Doce State Park (RDSP). Located in the southeastern portion of Minas Gerais State, in southeastern Brazil, the Park has an area of approximately 36,000 ha (IEF, 2021). The RDSP houses a great diversity of fauna and an abundance of natural resources, such as rivers, natural lakes, and a high‐quality forested area, factors that are often positively associated with the presence of jaguars (Boron et al., 2020; de la Torre et al., 2017; Lavariega et al., 2020; Santos et al., 2019). These characteristics reinforce the important role of RDSP in jaguars and jaguar prey conservation. Thus, knowing the jaguar abundance in RDSP would contribute to evaluate the existence of a potential jaguar conservation unit (JCU) in the region and provide a baseline for management actions to recover the species across the Atlantic Forest.

Management strategies to secure the last remaining jaguar populations can be evaluated by monitoring changes in jaguar density and exploring factors influencing jaguar's habitat use within the forest remnants. For example, evidence for a decrease in the density/abundance of jaguars combined with factors influencing the species habitat use within the RDSP may help to identify potential threats to the population which can be vital for planning actions to increase connectivity and build safe landscapes among the current highly isolated remaining populations. Indeed, for the Atlantic Forest ecosystem, ongoing habitat loss, fragmentation, and poaching have limited mammal species movements between natural habitat patches (Bogoni et al., 2018). Moreover, RDSP is mostly surrounded by human‐related habitat features such as cities, *Eucalyptus* plantations, and pastures, which have already been reported as negatively associated with the occurrence of jaguars (de la Torre et al., 2017; Xavier da Silva et al., 2018). Thus, monitoring the status of the jaguar population in the region would be vital to increase knowledge of the species' long‐term persistence in the Atlantic Forest remnants.

We aimed to (a) determine the jaguar density and abundance in RDSP, (b) quantify temporal population fluctuations, and (c) evaluate the influence of habitat features and human‐altered habitats on the occupancy and detection probabilities of jaguars in the Park. Jaguar movement may be affected spatially due to habitat characteristics and temporarily due to seasonal fluctuations (e.g., food resource) and human presence, which may influence detection probability (Bailey et al., 2004; Gu & Swihart, 2004; Morato et al., 2016). We expected probabilities of occupancy and detection of jaguars to be positively influenced by (a) the proximity to rivers and lakes (Boron et al., 2020), (b) increasing distances from cities, *Eucalyptus* plantations, and pastures (de la Torre et al., 2017; Xavier da Silva et al., 2018), and (c) higher numbers of prey records (Santos et al., 2019). We also expected higher detection probabilities during the dry season, because lower levels of rainfall during the dry season would promote less availability of prey species and thus maximize jaguar movement in searching for prey.

## 2 MATERIALS AND METHODS

### 2.1 Study area

**FIGURE 1:**
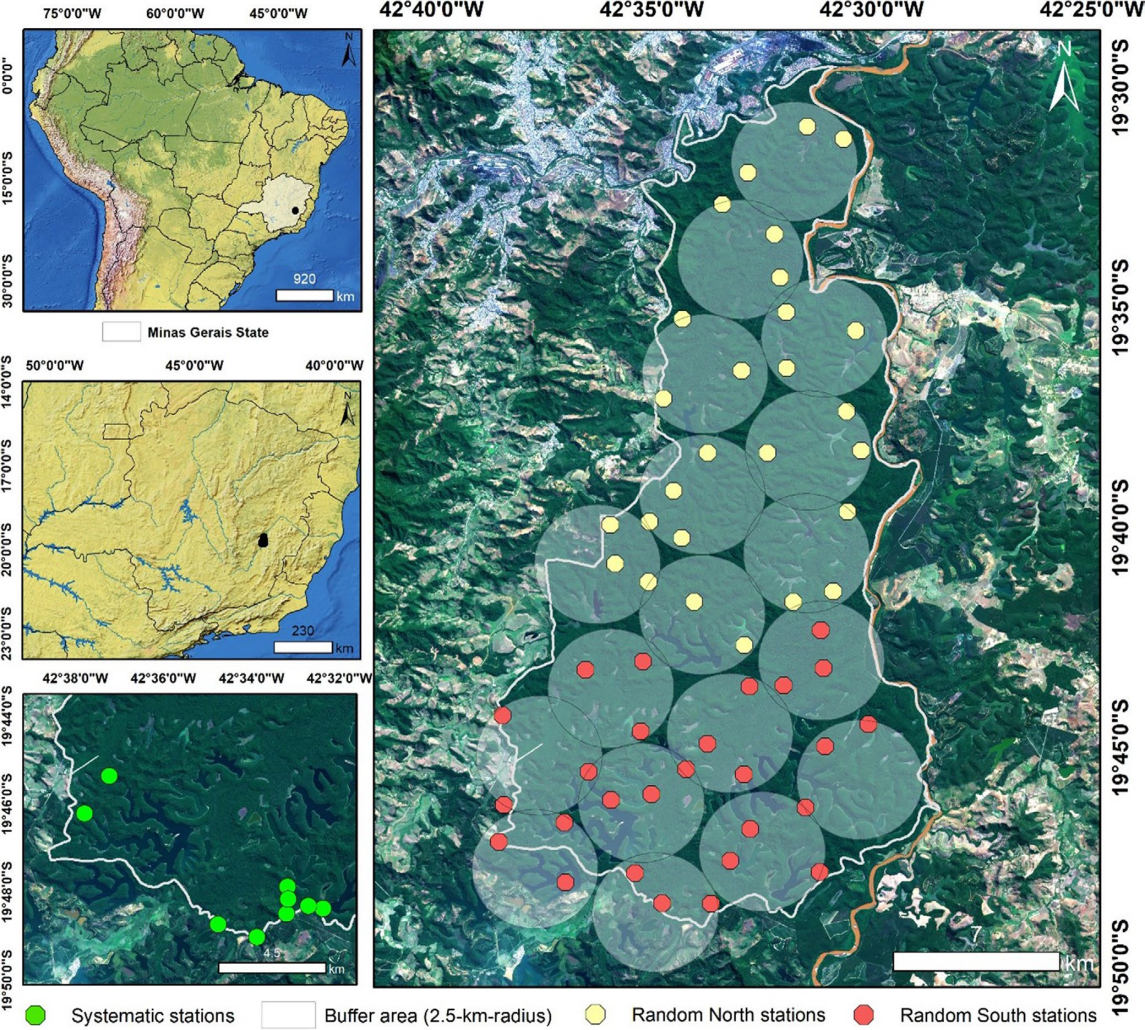
Location of the Rio Doce State Park, State of Minas Gerais, southeastern Brazil. The yellow and red grey dots represent the locations of the sampling stations used during the random sampling design (right), used to access the occurrence of jaguars. The green dots represent the locations of the sampling stations used during the systematic sampling design (bottom left), used to access fluctuations in the jaguar population. Geographic coordinate system: SIRGAS 2000 UTM Zone_23S. Source: IBGE (2019)

The RDSP is a strictly protected area (IUCN Category II) in the State of Minas Gerais, southeastern Brazil, representing one of the largest continuous remnants of Atlantic Forest in Brazil and the largest in the state of Minas Gerais (Gontijo & Britto, 1997). The RDSP represents an important area for maintenance of biodiversity in the Atlantic Forest (da Silva Junior et al., 2010). In addition to jaguars, the RDSP includes a variety of mammals such as pumas (*Puma concolor*), tapirs (*Tapirus terrestris*), collared peccaries (*Pecari tajacu*), northern‐muriquis (*Brachyteles hypoxanthus*), and giant armadillos (*Priodontes maximus*) (Keesen et al., 2016; da Silva Junior et al., 2010; Stallings et al., 1990). The RDSP has 42 natural lakes located mainly in the southern portion of the Park, three streams (Belém in the north, Turvo in the central area, and Mombaça in the south), and rivers Piracicaba and Doce bordering some areas of the Park (Figure 1). The vegetation is classified as submontane seasonal semideciduous forest (IBGE, 2002; Lino & Dias, 2004). The climate is humid subtropical, with two marked seasons: a rainy summer from October to March followed by a dry winter from April to September (Pereira et al., 2018). Human‐altered habitats around the Park are composed mainly of *Eucalyptus* plantations, pasture, and urban areas.

### 2.2 Survey design

We used a random survey design with a blocking arrangement of sampling units to estimate jaguar abundance and density as well as its occupancy and detection probabilities. By placing camera traps randomly in relation to animal movements, and sampling parts of the study area in proportion to their availability, we tried to avoid inflating or deflating encounter rates (Rowcliffe et al., 2013). We divided the study area into two sectors: north and south, each with nine 2.5‐km‐radius zones (buffers) covering the entire RDSP area. The area of circular zones corresponds to twice the smallest conservatively estimated home range size for female jaguars (10 km^2^ in a Central America tropical forest habitat) (Rabinowitz & Nottingham, 1986). We used a random point generator tool available in ArcGis 10.5 (create random points ‐ ESRI, 2016) to define three random sampling locations within each circular zone (Figure 1), which resulted in 27 camera trap stations (hereafter, stations) per sector. Minimum distance between stations was 1.35 km (average 1.87 ± 0.5 km, range 1.35–3.75 km), which represents a sampling intensity that should be enough to potentially detect all individuals in the population (Dillon & Kelly, 2007; Karanth & Nichols, 1998; Silver et al., 2004). Each station was composed of a pair of camera traps (Bushnell© Trophy Cam Natureview, Trophy Cam Standard, and Trophy Cam Essential—Kansas, USA) installed at 40–50 cm in height that were fixed to trees and facing each other. This design allowed us to typically record both flanks of a given animal, thereby facilitating individual identification by spots, marks, and scars (Karanth & Nichols, 1998). Cameras were programmed to record 10–30 s HD videos, with an interval of 60 s between videos. All cameras were programmed to operate simultaneously for 24 h/day, and we did not use baits or any other attractant. Due to lack of roads and access in remote areas of the RDSP, we opened 340 km of trails to access the designated stations. The data collection encompassed the dry (April 10–September 22, 2016) and rainy (October 17, 2016–April 17, 2017) seasons (Table S1). In each season, the survey of both sectors lasted a maximum of 120 days, which represents a short period in relation to the longevity of jaguar life span and migratory movements of individuals.

To monitor temporal fluctuations in jaguar density and abundance, we used a systematic survey design and performed two surveys (2017/18 and 2020). These surveys were conducted in the southern portion of the Park where more records of jaguars were obtained during the random survey. For the systematic design, nine stations were installed (Figure 1) along low‐traffic (<1 vehicle/day) unpaved roads which are rarely used and exclusive for research and maintenance (Figure 1). This sector represents approximately 14% of the study area (50 km^2^). The location of the stations was assigned to maximize the capture probability of carnivores (especially felids) by systematically selecting sites having direct evidence (e.g., tracks, scraps, scats) indicating their presence. Minimum distance between stations was 0.52 km (average 1.1 ± 0.58 km, range 0.52–1.84 km). This nonrandom approach aimed to increase jaguar detection probability and thus monitor any individuals with home ranges overlapping camera locations. Also, because jaguars have a large home range size, we assumed that this survey design was likely to detect any jaguar individual given it was present in the Park during the survey.

For our two systematic design surveys, the same stations were sampled during the dry and rainy seasons. For the 2017/18 survey, the sampling period encompassed dry (April 19 to September 30, 2017) and rainy (October 01, 2017, to January 23, 2018) seasons (Table S2). For the 2020 survey, the sampling period spanned dry (February 27 to September 30, 2020) and rainy (October 01 to December 12, 2020) seasons (Table S3).

### 2.3 Estimating density and abundance of jaguars

Jaguars were identified according to sex (when possible, visualization of genitalia), patterns of rosettes, and spots on both flanks (Karanth & Nichols, 1998; Noss et al., 2013). The identifications were performed by two observers independently (FCCA, JBP). We recorded individuals whose sex could not be confirmed as “unidentified” (N.I.). We discarded low‐quality records that were too blurry to allow clear observation of relevant features of jaguars (Figure S1). For each survey design (random and systematic), sampling effort was calculated by multiplying the number of sampling stations by the total number of days of operation of the stations, and capture success was calculated by dividing the number of jaguar records by the sampling effort and multiplying the result by 100.

We built the detection history for each individual through recording whether the individual was detected (1) or not (0) in each occasion, considering detections from all sampling stations. Due to the low number of jaguar records for both designs (random and systematic), we defined the sampling occasion length as 20 days, totaling 19 occasions for the random (2016/17) and 15 occasions for each systematic survey (2017/18 and 2020). We verified the assumption of population closure for both designs (random and systematic) between the dry and rainy seasons. For the systematic design specifically, we verified the closure separately for the 2017/2018 and 2020 survey periods. Thus, we defined each season as a different sampling session. We used the spatial capture and recapture models for open populations, available in the openCR package (Efford & Schofield, 2020) in Program R 3.5.2 (R Core Team, 2020) to verify the assumption of population closure for both random and systematic designs. For this, we used the spatial models of Jolly‐Seber‐Schwarz‐Arnason (JSSA) (Efford & Schofield, 2020; Schwarz & Arnason, 1996) that include the estimation of apparent survival (*φ*), probability of ingress (*b*), detection function (*λ*0), detection function scale (*σ*), and superpopulation density (*superD*) (Efford & Schofield, 2020). We built models allowing *φ* and *b* to be estimated between sessions (i.e., open population) or fixed (i.e., closed population – fixing *φ* = 1 and *b* = 0). Through the use of the Akaike information criterion adjusted for small sample sizes (AICc, Burnham & Anderson, 2002), we verified population closure for either the random design (ΔAICc = 5.78 for the next model with better support, in which *φ* and *b* were estimated), 2017/18 systematic design (ΔAICc = 4.26 for the next model with better support, in which *φ* and *b* were estimated), and 2020 systematic design (ΔAICc = 124 for the next model with better support, in which *φ* and *b* were estimated). Thus, we used spatially explicit capture and recapture models for closed populations (SCR) (Efford, 2004, 2019; Efford & Fewster, 2013) available in the secr package (Efford & Schofield, 2020) in Program R 3.5.2 (R Core Team, 2020).

We estimated the density of jaguars for the entire RDSP by using the data from the random survey design. Subsequently, we used the data from the systematic surveys for monitoring temporal jaguar population density fluctuations between the years 2017/18 and 2020. We conducted one analysis for the 2017/2018 survey period and another analysis for the 2020 survey period and, thus, estimated the model parameters of interest separately for each survey period. Spatially explicit methods for density estimates have some advantages over nonspatial methods as they consider heterogeneity in the detection of individuals and the geographic location of the records, which reduces the bias of estimates (Efford & Fewster, 2013). The SECR models estimate three parameters: the encounter rate (g0—probability of detecting an individual in the center of its living area), sigma (*σ*—distance, in meters, in which the detection probability decays from the center of the individual's living area—can serve as a proxy for the size of the living area of individuals), and a third derived parameter, density (estimation of individuals/100 km^2^) (Efford & Schofield, 2020; Espinosa et al., 2018). The encounter rate and sigma define the detection probability according to geographic locations (Efford, 2004; Efford & Schofield, 2020). For the three datasets (2016/17 random design, 2017/18 systematic design and 2020 systematic design), we fitted only the constant model (i.e., g0 ~ 1, sigma ~ 1, D ~ 1) to reduce the number of parameters estimated and thus minimize the bias of estimates as we had low individual recapture rates. Models were fitted using maximum likelihood estimation, assuming a Poisson distribution and the half‐normal detection functions (Efford, 2019; Efford & Schofield, 2020). We used as buffer dimension 4*σ* as suggested by Efford (2019) and Noss et al. (2013), resulting in a buffer of 15 km for the 2016/17 random design, 11 km m for the 2017/18 systematic design, and 11.2 km for the 2020 systematic design. From the spatial estimate of density generated by the models, we estimated the nonspatial abundance, multiplying the density by the size of the RDSP area.

### 2.4 Estimating the probabilities of occupancy and detection of jaguars

Here, we interpreted detection probability as the frequency (or intensity) of use of the occupied locations by jaguars (Dias et al., 2019; Massara et al., 2018) and occupancy probability as the probability a site *i* is occupied by the species (Mackenzie et al., 2002). To assess the influence of station‐level features on jaguar occupancy and detection probabilities, we measured the distance (m) between each station and the nearest river, lake, and human‐related habitats (urban areas, *Eucalyptus* plantations, and pasture), using a 2016 Sentinel‐2 satellite image (10‐m resolution) in ArcGIS 10.5 (ESRI, 2016) and SPRING 5.3. (Câmara et al., 1996). To explore the influence of prey availability on jaguar occupancy, we recorded the number of potential prey by summing all independent records of prey species at each station. To minimize dependence among records, we only included records of the same prey species that had an interval of one hour between them. We considered as potential prey for jaguars those species that had been previously recorded as having high overlap in activity patterns with jaguars in RDSP (Arrais, 2019) and that are also part of jaguar prey items according to the available literature (Seymour, 1989): capybaras (*Hydrochoerus hydrochaeris*), deer (*Mazama* spp.), tapirs (*Tapirus terrestris*), and peccaries (*Pecari tajacu*). We also constructed three covariates that varied for each station and sampling occasion to model detection probability only: the number of independent detections of potential prey species; the number of days the cameras were operational; and season as a categorical covariate (dry = 0 and rainy = 1) (Table 1). We evaluated for correlation among covariates using the Pearson correlation test to exclude highly correlated covariates (*r* ≥ .6) through the R 3.5.2 program (R Core Team, 2020; Wang et al., 2019), but none were highly correlated (Table S7).

**TABLE 1:** Covariates used to model the occupancy and detection probabilities of jaguars in the Rio Doce State Park, State of Minas Gerais, southeastern Brazil

Covariates	Mean value	Range value (min.–max.)	Parameter
Distance to the nearest river (m)	2106.87	169.71–11,948.36	*ѱ*, *p*
Distance to the nearest lake (m)	1292.38	0.00–3977.24	*ѱ*, *p*
Distance to human‐altered habitats (m)	2223.77	127.28–5287.15	*ѱ*, *p*
Days of active camera traps	12.82	0.00–20.00	*p*
Number of prey	25.15	0.00–221.00	*ѱ*
Detection of prey	2.54	0.00–54.00	*p*

Note

Days of active camera traps and detection of prey were measured for each occasion (11 occasions in total) of each station, and a weighted average was used among all stations in the mean value field.

We estimated the probabilities of occupancy and detection of jaguars by using the data from the random survey design of 2016/17. We combined detections into 20‐day periods (sampling occasions) to compose jaguar detection histories for each station and season. Thus, we generated a total of 11 occasions (six related to the dry and five related to the rainy seasons, respectively). First, we evaluated the premise of population closure using dynamic occupancy models, which allowed the parameters of colonization (gamma) and extinction (epsilon) of the stations between seasons to be estimated (MacKenzie et al., 2003). Two models were constructed, in which (a) the parameters of colonization and extinction were either estimated (open population; alternative hypothesis) or (b) fixed to zero (closed population; null hypothesis) (Massara et al., 2018; Nagy‐Reis et al., 2017). Using the AICc (Burnham & Anderson, 2002), both models were equally supported (ΔAICc = 0.08 for the closed population hypothesis), revealing weak evidence for an open population as our null hypothesis (i.e., intercept‐only model) was supported by the data. Also, as our main objective was not to evaluate occupancy changes between seasons, we combined data from both seasons in a static occupancy modeling approach (single‐season) (Mackenzie et al., 2002). This modeling approach includes the estimation of two parameters: the probability of occupancy (ѱ), which is defined as the probability a site *i* is occupied by the species; and the probability of detection (*p*), which is defined as the probability of detecting the species at a station *i* at a time (or occasion) t, given it is occupied (Mackenzie et al., 2002).

We built the models using Program MARK (White & Burnham, 1999) and ranked candidate models using the AICc (Burnham & Anderson, 2002). For the construction of the models, we used the step‐down strategy of model selection (Lebreton et al., 1992). Using a fixed structure of a global model (containing all covariates) for ѱ, we built different model structures with only one covariate for *p*. Based on the best‐ranked models that contained the most likely covariates (ΔAICc ≤ 2) for *p*, we began to build different model structures with only one covariate for ѱ, fixing the most likely covariates for *p* in a single model structure. We used the maximum likelihood estimation methods incorporated in Program MARK (Burnham & Anderson, 2002; Mackenzie, 2006) to estimate the probabilities of occupancy and detection of jaguars in RDSP. A total of 31 models were constructed (Table 2). As we had model selection uncertainty (i.e., more than one model with ΔAICc ≤ 2), occupancy and detection probabilities of jaguars for the RDSP were obtained through the model‐averaged estimates (Burnham & Anderson, 2002). By using the occupancy estimates for each sampling station from the best‐ranked covariate(s) influencing jaguar occupancy probabilities (ΔAICc ≤ 2), we generated a predictive map of jaguar occupancy for the entire RDSP using the “kriging” interpolate method available in ArcGIS (ESRI, 2016). We evaluated for lack of independence (i.e., overdispersion) among sampling stations by performing the goodness‐of‐fit test (MacKenzie & Bailey, 2004) available in Program PRESENCE 2.12.36 (Hines, 2006). For this test, we used the model with the largest number of covariates in ѱ, and *p* containing the covariate that had better support through the step‐down approach. The test revealed independence between the sampling stations (χ^2^ = 271.98, *p* = .70, *ĉ* = 1.00).

**TABLE 2:** Model set used to assess the occupancy and detection probabilities of jaguars in the Rio Doce State Park, State of Minas Gerais, southeastern Brazil

Model	AICc	ΔAICc	AICc weights	Number of parameters	−2 log(*L*)
Modeling *p*					
Ψ(riv + lak + hum + prey), p(prey‐t)^a^	121.59	0.00	0.26	7	105.16
Ψ(riv + lak + hum + prey), p(.)^a^	122.17	0.58	0.19	6	108.38
Ψ(riv + lak + hum + prey), p(season‐t)^a^	122.28	0.69	0.18	7	105.84
Ψ(riv + lak + hum + prey), p(lak)^a^	123.06	1.47	0.12	7	106.63
Ψ(riv + lak + hum + prey), p(day‐t)^a^	123.17	1.58	0.12	7	106.74
Ψ(riv + lak + hum + prey), p(rivers)	124.36	2.77	0.07	7	107.92
Ψ(riv + lak + hum + prey), p(hum)	124.69	3.10	0.06	7	108.25
Modeling Ψ					
Ψ(lak), p(prey‐t)	115.18	0.00	0.13	4	106.36
Ψ(.), p(prey‐t)	115.87	0.70	0.09	3	109.39
Ψ(.), p(season‐t)	116.75	1.57	0.06	3	110.27
Ψ(prey), p(prey‐t)	116.77	1.60	0.06	4	107.96
Ψ(lak), p(season‐t)	116.80	1.63	0.06	4	107.99
Ψ(.), p(lak)	116.84	1.66	0.06	3	110.36
Ψ(prey), p(season‐t)	116.92	1.74	0.05	4	108.10
Ψ(lak), p(.)	116.96	1.78	0.05	3	110.48
Ψ(.), p(.)	116.97	1.79	0.05	2	112.73
Ψ(prey), p(.)	117.08	1.91	0.05	3	110.60
Ψ(.), p(day‐t)	117.57	2.39	0.04	3	111.09
Ψ(prey), p(lak)	117.63	2.45	0.04	4	108.81
Ψ(lak), p(day‐t)	117.64	2.47	0.04	4	108.83
Ψ(prey), p(day‐t)	117.75	2.57	0.03	4	108.93
Ψ(riv), p(prey‐t)	118.15	2.97	0.03	4	109.33
Ψ(hum), p(prey‐t)	118.20	3.02	0.03	4	109.38
Ψ(riv), p(lak)	118.98	3.81	0.02	4	110.17
Ψ(hum), p(lak)	118.99	3.82	0.02	4	110.18
Ψ(riv), p(season‐t)	119.05	3.87	0.02	4	110.23
Ψ(hum), p(season‐t)	119.08	3.91	0.02	4	110.27
Ψ(lak), p(lak)	119.14	3.96	0.02	4	110.32
Ψ(riv), p(.)	119.17	4.00	0.02	3	112.69
Ψ(riv), p(day‐t)	119.87	4.70	0.01	4	111.06
Ψ(hum), p(day‐t)	119.91	4.73	0.01	4	111.09
Ψ(riv + lak + hum + prey), p(prey‐t)	121.59	6.41	0.01	7	105.16
Ψ(riv + lak + hum + prey), p(.)	122.17	6.99	0.00	6	108.38
Ψ(riv + lak + hum + prey), p(season‐t)	122.28	7.10	0.00	7	105.84
Ψ(riv + lak + hum + prey), p(lak)	123.06	7.89	0.00	7	106.63
Ψ(riv + lak + hum + prey), p(day‐t)	123.17	8.00	0.00	7	106.74
Ψ(riv + lak + hum + prey), p(riv)	124.36	9.18	0.00	7	107.92
Ψ(riv + lak + hum + prey), p(hum)	124.69	9.51	0.00	7	108.25

Note

Models were constructed through a step‐down approach (see text for details). The models were ranked using the Akaike information criterion adjusted for small samples (AICc); riv = distance between the station and the nearest river, lak = distance between the station and the nearest lake, hum = distance between the station and the nearest human‐altered habitat, prey = number of potential prey of jaguars recorded at the stations, prey‐t = detection of potential prey of jaguars recorded in each sampling occasion at each station, season‐t = season in each sampling occasion, day‐t = number of days the camera traps were operational in each sampling occasion at each station.

^a^ Best ranked models for *p* in the first step (ΔAICc ≤ 2) that were considered when modeling Ψ during the step‐down modeling approach.

## 3 RESULTS

### 3.1 Density and abundance of jaguars

**FIGURE 2:**
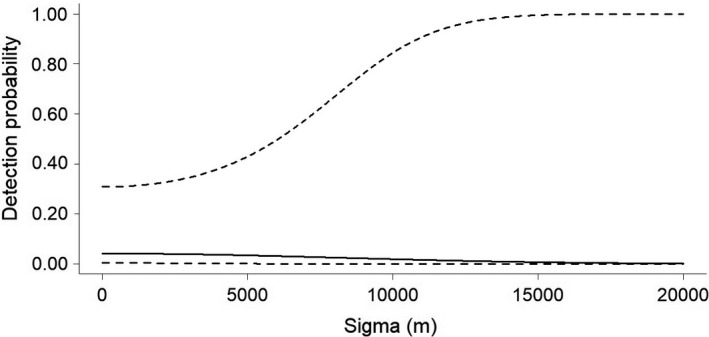
Detection probability (±95% CI – dashed lines) of jaguars as a function of the distance traveled from the center of the individuals' living area during random survey, in the Rio Doce State Park, State of Minas Gerais, southeastern Brazil

**FIGURE 3:**
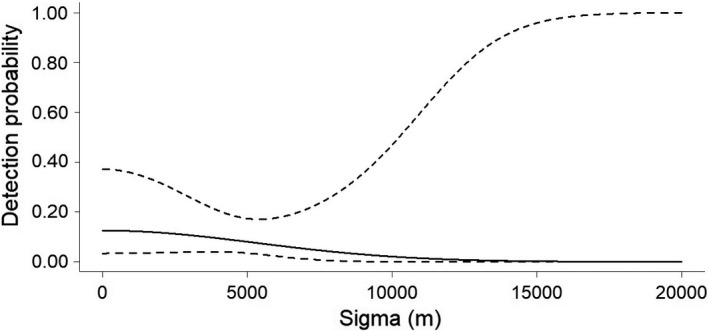
Detection probability (±95% CI – dashed lines) of jaguars as a function of the distance traveled from the center of the individuals' living area during 2017/18 systematic survey, in the Rio Doce State Park, State of Minas Gerais, southeastern Brazil

**FIGURE 4:**
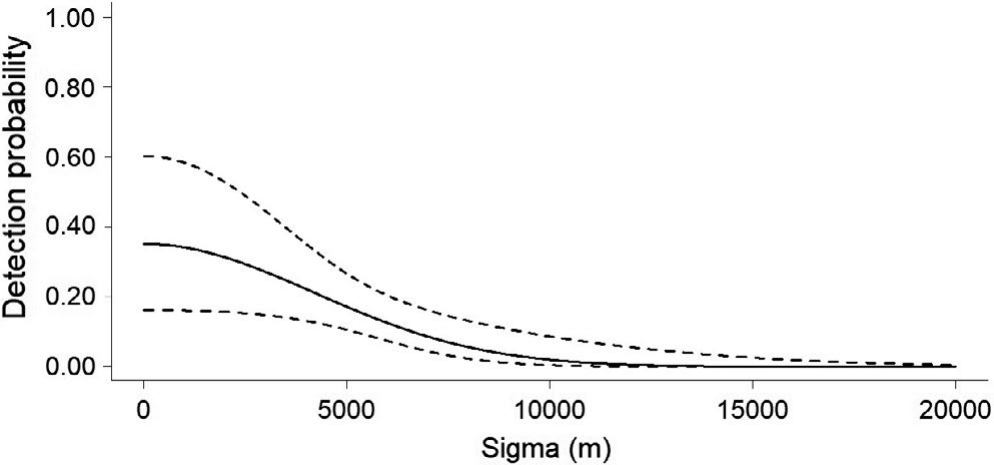
Detection probability (±95% CI – dashed lines) of jaguars as a function of the distance traveled from the center of the individuals' living area during 2020 systematic survey, in the Rio Doce State Park, State of Minas Gerais, southeastern Brazil

Considering both types of survey designs (random and systematic), we recorded a total of 10 jaguars (4 males, 4 females, 1 adult not identified (N.I) and 1 cub N.I.). We recorded only two males in the random survey. In the systematic surveys, we recorded three males, and all the females, one adult N.I., and one cub N.I. For the 2016/17 random survey design, a total of 7431 trap nights yielded 16 independent jaguar records (Table S4). The capture success rate was 0.18 captures per 100 trap nights. Our constant model provided an estimate of density (D) = 0.11 ± *SE* 0.28 individuals/100 km^2^ (95% CI = 0.02–0.69) and abundance (N) = 0.4 ± *SE* 0.99 individuals (95% CI = 0.06–2.49), g0 = 0.04 ± *SE* 0.05 (95% CI = 0.01–0.32), and *σ* = 8106.66 ± *SE* 15,828.91 m (95% CI = 3134.04–22,089.79 m) (Figure 2). For the 2017/18 systematic survey design, a total of 2089 trap nights yielded 52 independent identifiable jaguar records (Table S5). The capture success rate was 2.49 captures per 100 trap nights. From these records, we identified 3 jaguars, being 1 male (34.6% of records) and 2 females (65.4% of records). Our constant model provided an estimate of density (D) = 0.55 ± *SE* 0.45 individuals/100 km^2^ (95% CI = 0.13–2.26), abundance (N) = 1.97 ± *SE* 1.63 individuals (95% CI = 0.48–8.12), g0 = 0.13 ± *SE* 0.08 (95% CI = 0.03–0.37), and *σ* = 5292.21 ± *SE* 3788.28 m (95% CI = 1500.20–18,669.15 m) (Figure 3). For the 2020 systematic survey design, a total of 2329 trap nights yielded 82 independent identifiable jaguar records (Table S6). The capture success rate was 3.52 captures per 100 trap nights. From these records, we identified 9 jaguars, being 3 males (70.73% of records), 4 females (23.17% of records), 1 unidentified adult (4.9% of records), and 1 unidentified cub (1.22% of records). Our constant model provided an estimate of density (D) = 1.61 ± *SE* 0.6 individuals/100 km^2^ (95% CI = 0.79–3.27), abundance (N) = 5.78 ± *SE* 2.17 individuals (95% CI = 2.84–11.78), g0 = 0.35 ± *SE* 0.12 (95% CI = 0.16–0.6), and *σ* = 4180.44 ± *SE* 717.87 m (95% CI = 2993.04–5838.9 m) (Figure 4). All the 3 jaguars registered during the 2017/18 systematic survey were also registered during the 2020 systematic survey (one male and two females). Therefore, we registered 6 new individuals during the 2020 systematic survey design (Tables S5 and S6).

### 3.2 Occupancy and detection of jaguars

**FIGURE 5:**
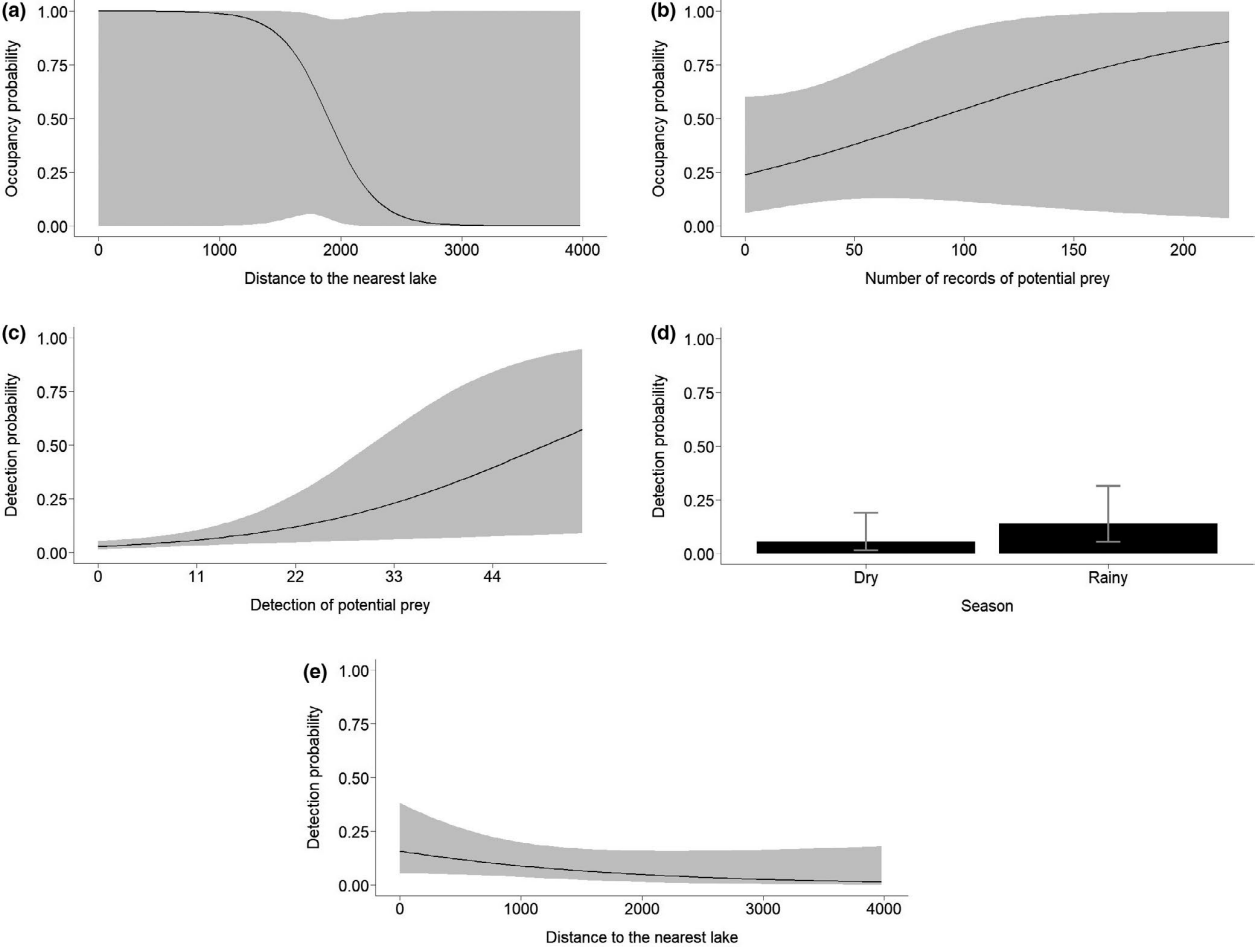
Effect of (a) distance (m) to the nearest lake and (b) number of potential preys recorded at each station on the occupancy probability (±95% CI) of jaguars. Effect of (c) detection of potential prey recorded in each sampling occasion at each station, (d) season in each sampling occasion, and (e) distance (m) to the nearest lake in the detection probability (±95% CI) of jaguars in the Rio Doce State Park, State of Minas Gerais, southeastern Brazil. Estimates and ±95% CIs were obtained from the best ranked model containing the covariate

**FIGURE 6:**
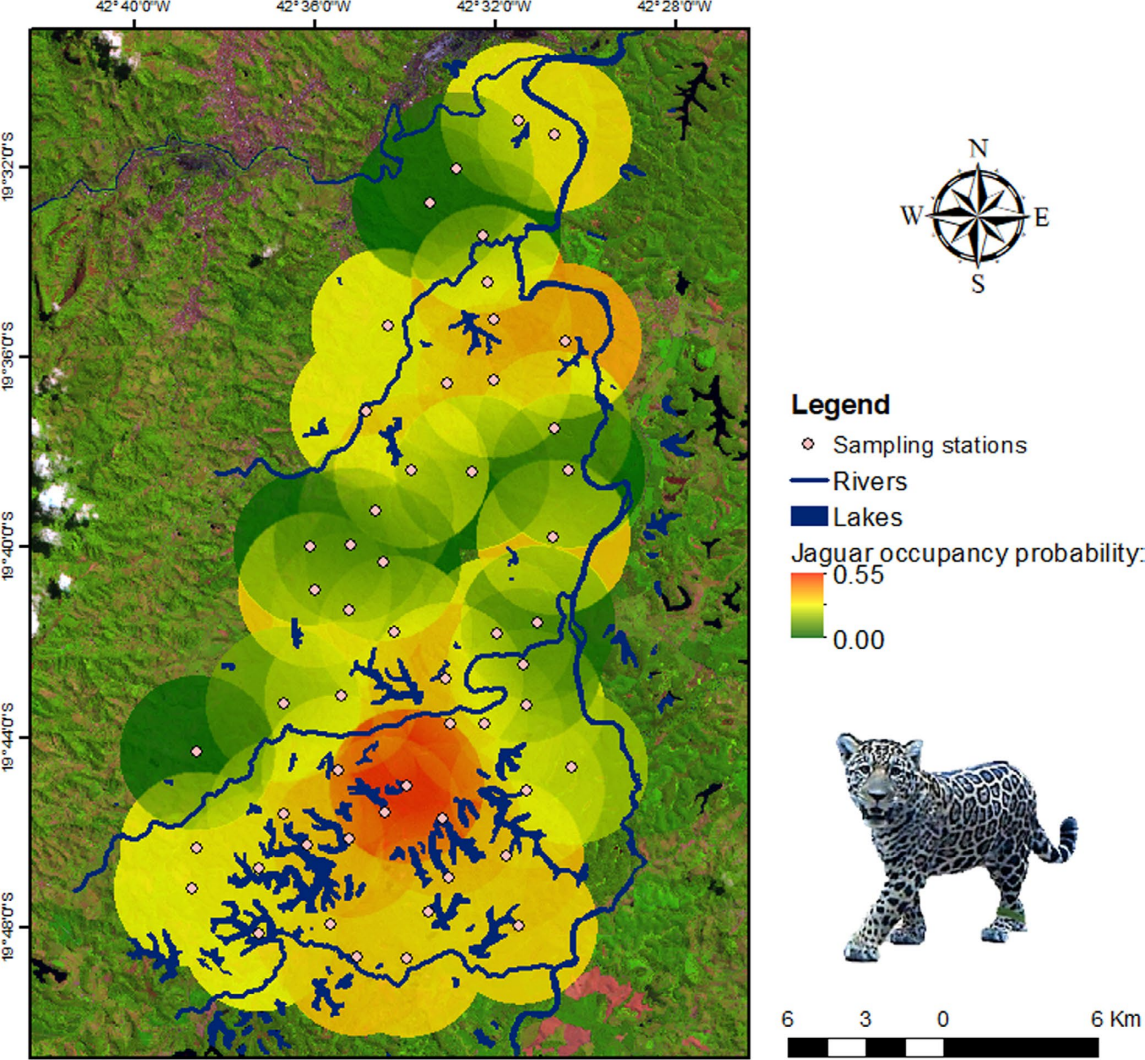
Interpolated sampling stations occupancy by jaguars in Rio Doce State Park, State of Minas Gerais, southeastern Brazil, during the random design survey. The lighter buffers around stations (white dots) represent higher jaguar occupancy probability, and the darker buffers represent lower jaguar occupancy probability. Geographic coordinate system: SIRGAS 2000 UTM_Zone_23S. Source: IBGE (2018)

We detected jaguars at 5 (out of 54) sampling stations (naïve occupancy = 0.09). We had more surveyed days in average during the rainy season (dry season/occasion = 9.9, *SE* = 0.36; rainy season/occasion = 16.4, *SE* = 0.34). From the model‐averaged estimates, $\hat{\Psi }$ = 0.40 (*SE* = 0.29), and $\hat{p}$ = .08 (*SE* = 0.07). According to model ranking (ΔAICc ≤ 2), the jaguar occupancy probability was positively associated with smaller distances from lakes and with the number of records of potential prey (Tables 2 and 3; Figure 5a,b), mostly in the southern region of RDSP (Figure 6). The jaguar detection probability (or intensity of use) was positively associated with prey detection, the rainy season, and smaller distances from lakes (Tables 2 and 3; Figure 5c–e). Because the null (intercept‐only) model structure was supported by our data when modeling either Ψ or *p* (Table 2), these covariates had only a slight influence on jaguar occupancy and detection probabilities.

**TABLE 3:** Estimates of *β* parameters for the covariates used to model the occupancy and detection probabilities of jaguars in the Rio Doce State Park, State of Minas Gerais, southeastern Brazil

Covariates	*β* parameters			
	Estimate	*SE*	LCI (95%)	UCI (95%)
Occupancy (Ψ)				
Distance to the nearest lake	**−0.005**	0.006	−0.017	0.007
Number of prey	**0.013**	0.013	−0.012	0.039
Distance to the nearest river	−0.00006	0.0003	−0.0006	0.0005
Distance to human‐altered habitats	−0.0001	0.0003	−0.0007	0.0004
Detection (*p*)				
Detection of prey	**0.07**	0.03	0.02	0.13
Season^a^	**1.00**	0.69	−0.34	2.35
**Distance to the nearest lake**	**−0.0007**	0.0004	−0.0015	0.0002
Days of active camera traps	0.08	0.07	−0.06	0.22
Distance to the nearest river	−0.0002	0.0003	−0.0003	0.0007
Distance to human‐altered habitats	0.00007	0.0002	−0.0003	0.0005

Note

The estimates of *β* parameters illustrate the influence of each covariate (positive or negative) on the model parameter and were obtained from the most parsimonious model containing the covariate. The estimates of *β* in bold represent the covariates that had the greatest support for the occupancy and detection of jaguars (ΔAICc ≤ 2). *SE* = standard error; LCI = lower confidence interval; UCI = upper confidence interval. Distance to the nearest lake: distance between the station and the nearest lake; number of prey: number of potential preys of jaguars recorded at the stations; distance to the nearest river: distance between the station and the nearest river; distance to human‐altered habitats: distance between the station and the nearest human‐altered habitat; detection of prey: detection of potential prey of jaguars recorded in each sampling occasion at each station; season: season in each sampling occasion; days of active camera traps: number of days the camera traps were operational in each sampling occasion at each station.

^a^ Beta values in reference to the rainy season.

## 4 DISCUSSION

Estimating density of large carnivores such as jaguars is challenging because they are generally wide‐ranging, occur at low densities and are elusive (Sollmann et al., 2011). The task is particularly challenging when this species is investigated within one of the most endangered major biomes worldwide, the Atlantic Forest. This biome is under current conditions of severe habitat alteration and fragmentation (Grelle et al., 2021). In our study, jaguars occurred at low density within the entire RDSP area (0.11 ind./100 km^2^ according to the estimates from the random survey design). Our estimates of jaguar density were the lowest obtained for the species across the entire Atlantic Forest (Paviolo et al., 2016; Srbek‐Araujo & Chiarello, 2017). We believe we have documented a decline of jaguar population between 2012 and 2015 based on a reduction of opportunistic records on cattle predation around RDSP while surveying ocelots in this conservation unit (Widmer et al., 2016). Although this hypothesis is rather speculative because we do not have jaguar density estimates from previous studies in the area, this possible jaguar population decline is an indicative of the importance of continuous monitoring of the population to fully understand its dynamics processes. The Atlantic Forest has undergone a long‐time history of poaching (Chiarello, 2000; Cullen et al., 2000; Paviolo et al., 2009) that has caused local extinction and affected the abundance and behavior of medium‐ and large‐sized mammal species (Chiarello, 2000; Di Bitetti et al., 2008; Paviolo et al., 2008). It is likely that jaguars in RDSP were depressed due to intense poaching in the surroundings of the Park and its borders, as reported for other forested regions (Romero‐Muñoz et al., 2019). In 2012, security in RDSP was controlled by a fixed company of environmental policy of the State of Minas Gerais comprising c. 16 men equating to 4.4 man per 100 km^2^ (FCCA, pers. observ.). In the following years, the company suffered from events of men being transferred to other regions of the State, and by the year 2020, there were no more fixed representatives of the policy within the RDSP. The diminished security personnel to control poaching suggests that jaguars may have been killed in the region in retaliation to livestock predation, thus contributing to the low density herein obtained for the entire RDSP. Although this hypothesis is rather speculative and not empirically grounded, ancillary data collected on events of jaguar predation on livestock in the surroundings of RDSP, the presence of poachers within RDSP and the lack of security to control poaching (FCCA, pers. observ.), give some support to this population trend.

As was expected based on previous studies with mammalian carnivores, jaguars were detected more often along trails and unpaved roads in the southern region of RDSP where our systematic survey was conducted. It is well known that some carnivores prefer roads or game trails for traveling (Carbone et al., 2001; Srbek‐Araujo & Chiarello, 2017) meaning that our observed increased capture and recapture rate on roads can represent an advantage for improving population density estimates. However, systematic survey designs can lead to biased or incomplete sampling if sampling locations (roads, trails) are restricted to specific areas or if animals differ in their probability of encounter (Cusack et al., 2015).

Although we did not aim at comparing the outcomes of the random sampling design with those of the systematic design, our results based on the 2017/18 systematic survey revealed that jaguar density estimate was slightly higher than that using the 2016/17 random survey design, which covered the entire RDSP. However, this increase may be related only to methodological differences in survey designs and not necessarily to an increase in abundance/density of jaguars between these periods. If we compare the confidence intervals of the model parameter estimates between surveys, we can conclude that both survey designs generated similar jaguar densities. It is important to highlight here that when the detection probability of elusive and rare species, such as jaguars, is low (especially ≤0.10) and each individual in the population is detected less than 2.5 times, capture–recapture models may generate imprecise abundance and density estimates, which may explain the large confidence intervals of our estimates regardless the survey design (Gerber et al., 2014). However, low detection probabilities are common among carnivore studies worldwide and our detection probability estimates are comparable to estimates reported by other studies ($\bar{p}$ = .17, range 0.02–0.79; Foster & Harmsen, 2012). Thus, even though the jaguar abundance/density estimates between these two periods were similar, with some differences regarding the presence of two females in the 2017/18 surveys that were not previously detected in the random survey, it is more likely that this difference might have occurred because of differences in the detectability between sampling designs and the fact that jaguars did prefer to use the southern portion of the RDSP. The southern region where the systematic survey was conducted represents approximately 14% of the study area has many water sources throughout the year as well as unpaved roads with very restricted traffic which may facilitate migratory movements of individuals. Thus, although the systematic survey design was not conducted in the entire Park and it did not account for potential detection differences between individuals and habitats, it revealed to be efficient for monitoring the jaguar population, especially concerning the movements of individuals in and out the Park.

Using the systematic survey design to monitor temporal fluctuations, jaguar density and abundance in RDSP changed significantly from 2017/18 to 2020. For the 2020 systematic survey, the number of jaguars was three times greater than that from the 2017/18 systematic survey. Thus, even under the worrisome regional status for the conservation of medium‐ and large‐bodied mammals in the Atlantic Forest, we found evidence of an established jaguar population in RDSP that includes several males and females and at least one jaguar cub. Our results suggest the presence of an increasing, reproductive, resident jaguar population in the study area (Andresen et al., 2012; De Angelo et al., 2013; Hidalgo‐Mihart et al., 2019; Patterson et al., 2004). One male and two females are considered residents because they were consistently detected in the same area at least 5 years in a row, and one of these females was observed with a cub (Barlow et al., 2009).

Changes in local jaguar density and abundance were mainly caused by changes in the number of adult animals and not in the number of nonbreeding animals represented by cubs or juveniles. The large fluctuation in the number of new animals was probably due to the immigration of adults into the population. With the COVID‐19 pandemic, the RDSP was closed for tourists and researchers for most of the 2020 year. The total lack of movement of people inside the RDSP may have contributed to the immigration movements of jaguars. Most of the RDSP surrounding areas are comprised of small patches of forest, being the RDSP the most preserved large patch of forest in the region. We believe that some jaguar individuals presented in the surroundings of the RDSP not detected in the surveys of 2016/17 and 2017/18 may have taken advantage of a population below the carrying capacity of RDSP and the absence of human activities within the park to move into RDSP, thus increasing the local population. A high‐quality forested area, with an abundance of water and wild prey, may have become more attractive to transient jaguars to immigrate to RDSP. The occurrence of large carnivores in anthropogenic landscapes adjacent to protected areas has been increasingly reported worldwide. For instance, leopards (*Panthera pardus*) and tigers (*Panthera tigris*) have been recorded living successfully in human‐dominated landscapes (Abade et al., 2018; Athreya et al., 2016; Kshettry et al., 2020) and in some cases breeding in those landscapes (Athreya et al., 2015). Jaguars in RDSP were reported using human‐dominated landscapes, as showed by two resident individuals that were monitored by GPS collars and spent most of their time outside the RDSP boundaries (Arrais, 2019). Thus, our assumption that some individuals were presented in the surroundings of the RDSP but not detected in the surveys of 2016/17 and 2017/18 and have immigrated to RDSP in 2020 probably holds true.

Our results showed that jaguar occupancy was low in RDSP (0.40). This is not surprising given the low density and large home ranges of jaguars and large carnivores in general, such as snow leopards (*Panthera uncia*), leopards, and tigers (Alexander et al., 2016; Sollmann et al., 2012; Strampelli et al., 2018; Wang et al., 2018). However, our occupancy estimate was lower than most of those reported for other protected forested sites in Central America, northern regions of the Amazon forest (Santos et al., 2019), and across the Atlantic Forest (Santos et al., 2018) and similar to those reported for forested sites within agricultural landscapes (Boron et al., 2020). Also, our occupancy estimate may be even lower than that we found, as our low detection probability estimate (0.08) may have generated a biased high jaguar occupancy estimate (Mackenzie et al., 2002) for the RDSP.

This low occupancy estimate may suggest that human‐altered modified landscapes may have a negative influence on jaguar occupancy. However, contrary to our expectations, increasing distances from cities, *Eucalyptus* plantations, and pastures did not influence jaguar occupancy probabilities in RDSP. On the other hand, according to our expectations, landscape features such as proximity to lakes and number of prey records had a slight positive effect on occupancy of jaguars. Although weak, we cannot ignore the positive effect of the presence of prey and proximity to lakes on jaguar occupancy in RDSP. These results indicate that the most suitable habitats for jaguars are in the southern region of the RDSP, where most of the lakes are located and also apparently most of the potential jaguar preys. The association of jaguars to water habitats has long been documented (Azevedo & Verdade, 2012; Figel et al., 2019; Ramalho et al., 2021) and jaguars in RDSP seemed to follow this pattern.

As stated, the overall jaguar detection probability was low (0.08) in RDSP. As predicted, jaguar detection was slightly higher at occupied sites with relatively more records of prey and near lakes, being mostly located in the southern region of the RDSP. Thus, this result suggests that detectability was lower in occupied stations in the northern region of RDSP, indicating that jaguars could be using with less intensity locations with less availability of permanent water sources and where the jaguar preys are rare. Contrary to our expectations, jaguar detection probabilities were higher during the rainy season. The fact that most of the lakes are located in the southern region of the Park might shed some light on the influence of season on detection probabilities. It is already known that the distribution of some species of herbivores is more homogeneous during the rainy season when surface water is more abundant due to formation of temporary water pools in addition to the permanent water sources, particularly in semiarid ecosystems (Davidson et al., 2013; Valeix et al., 2010). It is also known that prey productivity increases during rainy seasons, thus scaling carnivore population density (Carbone & Gittleman, 2002; Santos et al., 2019). Although the climate is humid in RDSP, the dry winters may represent substantial decreases in precipitation and lack of surface water within the Park. The 42 lakes and three streams within RDSP represent most of the permanent water sources, but during the dry seasons, the levels of some of them diminish significantly. It is reasonable to infer that the most important prey species for jaguars would be more homogeneously distributed during the rainy seasons than during the dry seasons, when prey would be aggregated near lakes. In addition, some carnivore species tend to increase their predation rates upon medium‐ and large‐bodied prey and restrict their movements near water sources (Valeix et al., 2010).

## 5 CONCLUSIONS

Our work represents one of the most comprehensive population estimate and investigation of factors influencing jaguar habitat use in a protected area of the Atlantic Forest. Because population surveys had not previously been attempted in the entire RDSP, a relatively large effort was expended to detect jaguars and maximize sample sizes. When compared with other jaguar density estimates for the Atlantic Forest, the low‐density estimates herein reported could have resulted from methodological and analytical differences. We should emphasize that our design represented an attempt to estimate jaguar density within the entire study area by placing camera traps randomly in relation to animal movements. Most jaguar density estimates across the Atlantic Forest are based on data collected from camera traps placed along roads and main trails where detection probabilities tend to be greater, thus inflating encounter rates (Paviolo et al., 2008, 2016; Rowcliffe et al., 2013; Srbek‐Araujo & Chiarello, 2017), then our abundance estimates based on the systematic survey design may still be comparable with these latter studies. In addition, our sampling effort (number of cameras and trap nights) was similar or even greater than that of other studies. Thus, the random arrangement of cameras and great sampling effort suggest that our jaguar density estimate was reliable.

It was evident from our data that jaguars were more detected along trails and unpaved roads in the region with better habitat quality of the Park and that transient individuals were only detected in this region (i.e., southern). Monitoring attempts that identify the preferred habitat sectors of a population, such as the southern portion of RDSP, have more power to detect changes than comparable efforts tracking total abundance, herein represented by the entire RDSP (Barlow et al., 2009). We suggest that studies attempting to estimate habitat use and abundance should primarily focus on a random approach of the entire study area and then on the more frequently used areas to monitor population changes, as performed here. Thus, our density estimates and occupancy probabilities represent not only a temporal snapshot assessment of jaguar population estimates and habitat use, but rather a first attempt to track population change over time across the Atlantic Forest. The possible immigration movement of adult animals into the RDSP population and the more intense use of the southern portion of the Park suggest that unprotected and human‐modified areas are crucial for jaguar conservation across the Atlantic Forest. Nonetheless, further evaluation is needed using spatial replicates set within and outside Park boundaries to reliably and efficiently identify jaguar population sources and changes in abundance over time and evaluate potential threats represented by human‐related activities.

Jaguars in RDSP will greatly benefit from attempts to implement a management program that would include: (a) re‐establishing gene flow between isolated jaguar populations across the Atlantic Forest, particularly the one at Linhares‐Sooretama block in the State of Espírito Santo (Srbek‐Araujo & Chiarello, 2017); (b) systematically monitoring jaguar populations and their prey array, and (c) increasing security personnel to control poaching within and outside protected areas in the biome. Our work contributes to a growing awareness of the potential conservation value of a protected area in a human‐dominated landscape as one of the last strongholds for jaguars across the Atlantic Forest and helps to monitor jaguar metapopulations.

## CONFLICT OF INTEREST

The authors declare that they have no conflict of interest.

## AUTHOR CONTRIBUTIONS

**Fernando Cesar Cascelli de Azevedo:** Conceptualization (lead); data curation (equal); formal analysis (equal); funding acquisition (lead); investigation (equal); methodology (equal); project administration (lead); resources (lead); supervision (lead); validation (lead); visualization (lead); writing – original draft (lead); writing – review & editing (lead). **Juliana Benck Pasa:** Formal analysis (equal); investigation (equal); writing – original draft (supporting); writing – review & editing (supporting). **Ricardo Corassa Arrais:** Data curation (supporting); investigation (supporting); writing – original draft (supporting); writing – review & editing (supporting). **Rodrigo Lima Massara:** Data curation (supporting); formal analysis (equal); investigation (supporting); validation (equal); writing – original draft (equal); writing – review & editing (equal). **Cynthia Widmer Azevedo:** Conceptualization (equal); data curation (equal); formal analysis (supporting); funding acquisition (lead); investigation (supporting); project administration (lead); writing – original draft (supporting); writing – review & editing (supporting).

## Supporting information

Supplementary Material

Fig S1

## Data Availability

All data from the manuscript are provided in the supplementary material.
