# Supplementary material for: Density and habitat use of one of the last jaguar populations of the Brazilian Atlantic Forest: Is there still hope?
Source: Ecol Evol. 2022 Jan 15;12(1):e8487. doi: 10.1002/ece3.8487 (PMC8809435; doi:10.1002/ece3.8487)
Supplement: Supplementary file 1 — Supplementary Material [file ECE3-12-e8487-s002.docx]

**Supplementary Material**

**Table S1.** Sampling period of each station during the random design, in Rio Doce State Park, State of Minas Gerais, southeastern Brazil. The stations were sampled during one dry and one rainy season, from April 2016 to April 2017.

| **Sampling period - Random design** | | | | | | | | | | | | | | |
| --- | --- | --- | --- | --- | --- | --- | --- | --- | --- | --- | --- | --- | --- | --- |
| **Sampling** |  | **Dry** | **Dry** | **Dry** | **Dry** | **Dry** | **Dry** | **Rainy** | **Rainy** | **Rainy** | **Rainy** | **Rainy** | **Rainy** | **Dry** |
| **station** | **Region** | **apr/2016** | **may/2016** | **jun/2016** | **jul/2016** | **aug/2016** | **sep/2016** | **oct/2016** | **nov/2016** | **dec/2016** | **jan/2017** | **feb/2017** | **mar/2017** | **apr/2017** |
| 1 | North | apr 15 ---------------------- jun 23 | | |  |  |  |  |  |  | jan 24 ------------------------------ apr 17 | | | |
| 2 | North |  | may 3 ------- jun 15 | |  |  |  |  |  |  | jan 18 ------------------------------ apr 11 | | | |
| 3 | North | apr 15 ---------------------- jun 23 | | |  |  |  |  |  |  | jan 24 ------------------------------ apr 17 | | | |
| 4 | North | apr 14 ---------------------- jun 20 | | |  |  |  |  |  |  | jan 23 ------------------------------ apr 14 | | | |
| 5 | North |  | may 3 ------- jun 15 | |  |  |  |  |  |  | jan 18 ------------------------------ apr 11 | | | |
| 6 | North | apr 17 ---------------------- jun 20 | | |  |  |  |  |  |  | jan 21 ------------------------------ apr 14 | | | |
| 7 | North | apr 13 ---------------------- jun 14 | | |  |  |  |  |  |  | jan 15 ------------------------------ apr 13 | | | |
| 8 | North | apr 13 ---------------------- jun 16 | | |  |  |  |  |  |  | jan 16 ------------------------------ apr 13 | | | |
| 9 | North |  | may 1 ------- jun 15 | |  |  |  |  |  |  | jan 20 ------------------------------ apr 12 | | | |
| 10 | North | apr 19 ---------------------- jun 22 | | |  |  |  |  |  |  | jan 20 ------------------------------ apr 12 | | | |
| 11 | North | apr 18 ---------------------- jun 22 | | |  |  |  |  |  |  | jan 25 ------------------------------ apr 16 | | | |
| 12 | North |  | may 1 ------- jun 15 | |  |  |  |  |  |  | jan 26 ------------------------------ apr 16 | | | |
| 13 | North | apr 30 ---------------------- jun 13 | | |  |  |  |  |  |  | jan 15 ------------------------------ apr 6 | | | |
| 14 | North | apr 11 ---------------------- jun 13 | | |  |  |  |  |  |  | jan 11 ------------------------------ apr 6 | | | |
| 15 | North | apr 11 ---------------------- jun 13 | | |  |  |  |  |  |  | jan 14 ------------------------------ apr 6 | | | |
| 16 | North | apr 13 ---------------------- jun 13 | | |  |  |  |  |  |  | jan 14 ------------------------------ apr 6 | | | |
| 17 | North |  | may 2 ------- jun 16 | |  |  |  |  |  |  | jan 19 ----------------------------- apr 10 | | | |
| 18 | North | apr 12 ---------------------- jun 14 | | |  |  |  |  |  |  | jan 13 ------------------------------ apr 7 | | | |
| 19 | North | apr 26 ---------------------- jun 24 | | |  |  |  |  |  |  | jan 25 ----------------------------- apr 15 | | | |
| 20 | North |  | may 2 ------- jun 16 | |  |  |  |  |  |  | jan 19 ----------------------------- apr 10 | | | |
| 21 | North | apr 26 ---------------------- jun 24 | | |  |  |  |  |  |  | jan 25 ----------------------------- apr 15 | | | |
| 22 | North | apr 28 ---------------------- jun 21 | | |  |  |  |  |  |  | jan 17 ------------------------------ apr 8 | | | |
| 23 | North | apr 27 ---------------------- jun 16 | | |  |  |  |  |  |  | jan 12 ------------------------------ apr 5 | | | |
| 24 | North | apr 12 ---------------------- jun 14 | | |  |  |  |  |  |  | jan 13 ------------------------------ apr 7 | | | |
| 25 | North | apr 10 ---------------------- jun 12 | | |  |  |  |  |  |  | jan 14 ----------------------------- apr 4 | | | |
| 26 | North | apr 11 ---------------------- jun 13 | | |  |  |  |  |  |  | jan 11 ------------------------------ apr 4 | | | |
| 27 | North | apr 25 ---------------------- jun 25 | | |  |  |  |  |  |  | jan 26 ----------------------------- apr 15 | | | |
| 28 | South |  |  |  | jul 1 ------- aug 28 | |  | oct 23 ------------------------------ jan 10 | | | |  |  |  |
| 29 | South |  |  |  | jul 10 ------- aug 28 | |  | oct 17 ------------------------------ jan 8 | | | |  |  |  |
| 30 | South |  |  |  | jul 14 ------- aug 28 | |  | oct 17 ------------------------------ jan 8 | | | |  |  |  |
| 31 | South |  |  |  | jul 11 ------- aug 25 | |  | oct 23 ----------------- dec 31 | | |  |  |  |  |
| 32 | South |  |  |  | jul 6 ------- aug 25 | |  | oct 23 ----------------- dec 31 | | |  |  |  |  |
| 33 | South |  |  |  | jul 5 ------- aug 28 | |  | oct 21 ------------------------------ jan 6 | | | |  |  |  |
| 34 | South |  |  |  | jul 3 ------- aug 29 | |  | oct 21 ------------------------------ jan 6 | | | |  |  |  |
| 35 | South |  |  |  | jul 5 ------- aug 29 | |  | oct 21 ------------------------------ jan 6 | | | |  |  |  |
| 36 | South |  |  |  | jul 3 ------- aug 29 | |  | oct 21 ------------------------------ jan 6 | | | |  |  |  |
| 37 | South |  |  | jun 30 ----------------- aug 31 | | |  | oct 18 ------------------------------ jan 7 | | | |  |  |  |
| 38 | South |  |  | jun 27 ----------------- aug 28 | | |  | oct 17 ------------------------------ jan 5 | | | |  |  |  |
| 39 | South |  |  | jun 27 ----------------- aug 27 | | |  | oct 16 ------------------------------ jan 7 | | | |  |  |  |
| 40 | South |  |  | jun 17 ----------------- aug 25 | | |  | oct 18 ------------------------------ jan 7 | | | |  |  |  |
| 41 | South |  |  |  | jul 6 ------- aug 25 | |  | oct 23 ------------------------------ jan 10 | | | |  |  |  |
| 42 | South |  |  |  | jul 1 ------- aug 25 | |  | oct 23 ----------------- dec 31 | | |  |  |  |  |
| 43 | South |  |  |  | jul 15 ------- aug 27 | |  | oct 16 ------------------------------ jan 5 | | | |  |  |  |
| 44 | South |  |  | jun 28 ----------------- aug 27 | | |  | oct 16 ------------------------------ jan 5 | | | |  |  |  |
| 45 | South |  |  | jun 29 ----------------- aug 27 | | |  | oct 16 ------------------------------ jan 5 | | | |  |  |  |
| 46 | South |  |  |  | jul 12 ------- aug 26 | |  | oct 20 ------------------------------ jan 21 | | | |  |  |  |
| 47 | South |  |  | jun 30 ----------------- aug 27 | | |  | oct 19 ------------------------------ jan 10 | | | |  |  |  |
| 48 | South |  |  |  | jul 13 ------- aug 26 | |  | oct 20 ------------------ dec 20 | | |  |  |  |  |
| 49 | South |  |  | jun 27 ----------------- aug 28 | | |  | oct 16 ------------------------------ jan 5 | | | |  |  |  |
| 50 | South |  |  | jun 27 ----------------- aug 26 | | |  | oct 16 ------------------------------ jan 7 | | | |  |  |  |
| 51 | South |  |  | jun 12 ----------------- aug 28 | | |  | oct 18 ------------------------------ jan 7 | | | |  |  |  |
| 52 | South |  |  |  | jul 29 ----------------- sep 22 | | | oct 16 ------------------------------ jan 5 | | | |  |  |  |
| 53 | South |  |  | jun 28 ----------------- aug 27 | | |  | oct 16 ------------------------------ jan 5 | | | |  |  |  |
| 54 | South |  |  | jun 17 ----------------- aug 29 | | |  | oct 16 ------------------------------ jan 7 | | | |  |  |  |

**Table S2.** Sampling period of each station during the 2017/18 systematic design, in Rio Doce State Park, State of Minas Gerais, southeastern Brazil. The stations were sampled during one dry and one rainy season, from April 2017 to January 2018.

| **Sampling period - Systematic design 2017/18** | | | | | | | | | | | |
| --- | --- | --- | --- | --- | --- | --- | --- | --- | --- | --- | --- |
| **Sampling** |  | **Dry** | **Dry** | **Dry** | **Dry** | **Dry** | **Dry** | **Rainy** | **Rainy** | **Rainy** | **Rainy** |
| **station** | **Region** | **apr/2017** | **may/2017** | **jun/2017** | **jul/2017** | **aug/207** | **sep/2017** | **oct/2017** | **nov/2017** | **dec/2017** | **jan/2018** |
| 55 | South |  | may 14 ----------------------------------------------------------------------- nov 24 | | | | | | |  |  |
| 56 | South | apr 20 ----------------------------------------------------------------------------------------------------- dec 18 | | | | | | | | |  |
| 57 | South | apr 19 ------------------------------------------------------------------------------------------------------------------ jan 23 | | | | | | | | | |
| 58 | South | apr 19 ------------------------------------------------------------------------------------------------------------------ jan 26 | | | | | | | | | |
| 59 | South | apr 20 --- may 30 | | jun 4 ----------------------------------------------------------- nov 24 | | | | | |  |  |
| 60 | South | apr 20 --------------------------------------------------------------------------------------- nov 24 | | | | | | | |  |  |
| 61 | South | apr 20 --------------------------------------------------------------------------------------- nov 24 | | | | | | | |  |  |
| 62 | South | apr 20 --------------------------------------------------------------------------------------- nov 24 | | | | | | | |  |  |
| 63 | South | apr 20 --------------------------------------------------------------------------------------- nov 24 | | | | | | | |  |  |

**Table S3.** Sampling period of each station during the 2020 systematic design, in Rio Doce State Park, State of Minas Gerais, southeastern Brazil. The stations were sampled during one dry and one rainy season, from February 2020 to December 2020.

| **Sampling period - Systematic design 2020** | | | | | | | | | | | | |  |
| --- | --- | --- | --- | --- | --- | --- | --- | --- | --- | --- | --- | --- | --- |
| **Sampling** |  | **Rainy** | **Rainy** | **Dry** | **Dry** | **Dry** | **Dry** | **Dry** | **Dry** | **Rainy** | **Rainy** | **Rainy** | |
| **station** | **Region** | **feb/2020** | **mar/2020** | **apr/2020** | **may/2020** | **jun/2020** | **jul/2020** | **aug/2020** | **sep/2020** | **oct/2020** | **nov/20** | **dec/2020** | |
| 55 | South | feb 29 ----------------------------------------------------------------------------------------------------------------------- dec 12 | | | | | | | | | | |  |
| 56 | South | feb 29 ----------------------------------------------------------------------------------------------------------------------- dec 12 | | | | | | | | | | |  |
| 57 | South | feb 28 ------------------ apr 20 aug 3 ------------------------------------------dec 12 | | | | | | | | | | |  |
| 58 | South | feb 27 ---------------------------------------------- jul 28 | | | | | | aug 3 ------------------------------------------ dec 12 | | | | |  |
| 59 | South | feb 29 ------------------------------------------------------------------------------------------------------------------------ dec 12 | | | | | | | | | | |  |
| 60 | South | feb 29 ------------------------------------------------------------------------------------------------------------------------ dec 12 | | | | | | | | | | |  |
| 61 | South | feb 29 ------------------------------------------------------------------------------------------------------------------------ dec 12 | | | | | | | | | | |  |
| 62 | South |  |  |  |  |  |  | aug 4 ------------------------------------------ dec 12 | | | | |  |
| 63 | South | feb 29 ------------------------------------------------------------------------------------------------------------------------ dec 12 | | | | | | | | | | |  |

**Table S4.** Capture data of jaguars in Rio Doce State Park, State of Minas Gerais, southeastern Brazil, during the random design survey. This data was used to obtain jaguar density estimates through spatially explicit capture-recapture modelling. ID = individual identification.

| Session | ID | Occasion | Detector |
| --- | --- | --- | --- |
| 1 | #Male#01 | 7 | 32 |
| 1 | #Male#01 | 8 | 52 |
| 1 | #Male#01 | 11 | 38 |
| 1 | #Male#01 | 12 | 33 |
| 1 | #Male#01 | 12 | 38 |
| 1 | #Male#02 | 6 | 54 |
| 1 | #Male#02 | 10 | 44 |
| 1 | #Male#02 | 11 | 46 |

**Table S5.** Capture data of jaguars in Rio Doce State Park, State of Minas Gerais, southeastern Brazil, during the 2017/18 systematic design survey. This data was used to obtain jaguar density estimates through spatially explicit capture-recapture modelling. ID = individual identification.

| Session | ID | Occasion | Detector |
| --- | --- | --- | --- |
| 1 | #Male#01 | 2 | 65 |
| 1 | #Male#01 | 5 | 61 |
| 1 | #Male#01 | 7 | 56 |
| 1 | #Male#01 | 7 | 60 |
| 1 | #Male#01 | 8 | 61 |
| 1 | #Male#01 | 8 | 65 |
| 1 | #Male#01 | 8 | 59 |
| 1 | #Male#01 | 9 | 62 |
| 1 | #Male#01 | 11 | 65 |
| 1 | #Female#01 | 3 | 63 |
| 1 | #Female#01 | 3 | 73 |
| 1 | #Female#01 | 6 | 63 |
| 1 | #Female#01 | 7 | 65 |
| 1 | #Female#01 | 8 | 65 |
| 1 | #Female#02 | 6 | 63 |

**Table S6.** Capture data of jaguars in Rio Doce State Park, State of Minas Gerais, southeastern Brazil, during the 2020 systematic design survey. This data was used to obtain jaguar density estimates through spatially explicit capture-recapture modelling. ID = individual identification.

| Session | ID | Occasion | Detector |
| --- | --- | --- | --- |
| 1 | #Male#01 | 1 | 65 |
| 1 | #Male#01 | 2 | 61 |
| 1 | #Male#01 | 2 | 65 |
| 1 | #Male#01 | 3 | 61 |
| 1 | #Male#01 | 3 | 65 |
| 1 | #Male#01 | 4 | 65 |
| 1 | #Male#01 | 4 | 61 |
| 1 | #Male#01 | 5 | 65 |
| 1 | #Male#01 | 5 | 62 |
| 1 | #Male#01 | 5 | 63 |
| 1 | #Male#01 | 5 | 56 |
| 1 | #Male#01 | 6 | 56 |
| 1 | #Male#01 | 6 | 62 |
| 1 | #Male#01 | 7 | 61 |
| 1 | #Male#01 | 7 | 65 |
| 1 | #Male#01 | 9 | 73 |
| 1 | #Male#01 | 9 | 71 |
| 1 | #Male#01 | 9 | 60 |
| 1 | #Male#01 | 10 | 60 |
| 1 | #Male#01 | 11 | 73 |
| 1 | #Male#01 | 11 | 71 |
| 1 | #Male#01 | 11 | 65 |
| 1 | #Male#01 | 11 | 61 |
| 1 | #Male#01 | 13 | 61 |
| 1 | #Male#03 | 3 | 59 |
| 1 | #Male#04 | 5 | 59 |
| 1 | #Male#04 | 10 | 73 |
| 1 | #Male#04 | 10 | 71 |
| 1 | #Male#04 | 10 | 56 |
| 1 | #Male#04 | 13 | 73 |
| 1 | #Male#04 | 13 | 71 |
| 1 | #Male#04 | 14 | 73 |
| 1 | #Female#01 | 6 | 73 |
| 1 | #Female#01 | 10 | 73 |
| 1 | #Female#02 | 2 | 61 |
| 1 | #Female#02 | 4 | 62 |
| 1 | #Female#02 | 4 | 61 |
| 1 | #Female#02 | 7 | 73 |
| 1 | #Female#02 | 9 | 59 |
| 1 | #Female#03 | 5 | 65 |
| 1 | #Female#03 | 6 | 56 |
| 1 | #Female#03 | 7 | 61 |
| 1 | #Female#03 | 7 | 63 |
| 1 | #Female#03 | 9 | 73 |
| 1 | #NI#01 | 3 | 59 |
| 1 | #NI#01 | 4 | 59 |
| 1 | #Cub#01 | 9 | 60 |
| 1 | #Female#04 | 2 | 61 |

**Table S7.** Pearson’s correlation test between habitat covariates considered to model occupancy and detection probabilities of jaguars in Rio Doce State Park, State of Minas Gerais, southeastern Brazil. Covariates highly correlated (*r* > 0.6) were removed from the analysis (indicated with an asterisk). Rivers: distance between the station and the nearest river; lakes: distance between the station and the nearest lake; human-altered: distance between the station and the nearest human-altered habitat; number of days: number of days the camera traps were operational in each sampling occasion at each station; prey records: number of potential preys of jaguars recorded at the stations.

|  | **Rivers** | **Lakes** | **Human-altered** | **Number of days** | **Prey records** |
| --- | --- | --- | --- | --- | --- |
| **Rivers** | - | -0.14 | 0.34 | -0.19 | 0.07 |
| **Lakes** |  | - | -0.32 | 0.08 | -0.25 |
| **Human-altered** |  |  | - | -0.54 | 0.37 |
| **Number of days** |  |  |  | - | -0.36 |
| **Prey records** |  |  |  |  | - |


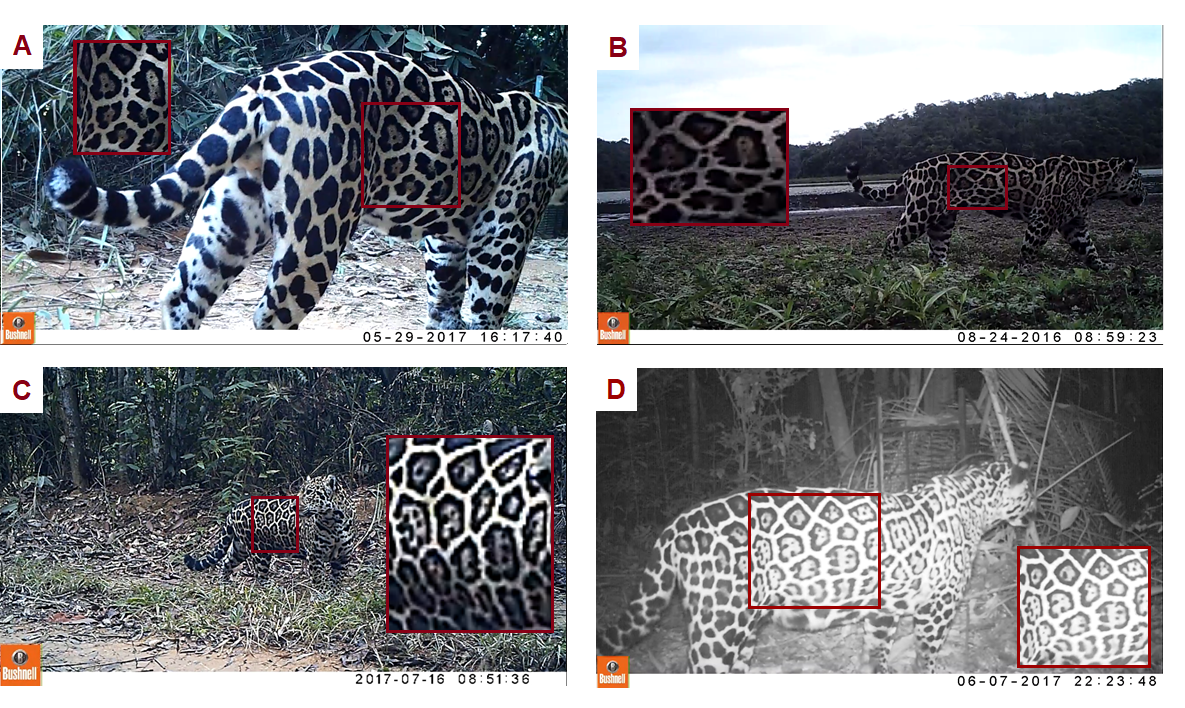


**Figure S1.** Examples of individual identification of jaguars in Rio Doce State Park,

State of Minas Gerais, southeastern Brazil. A and B are records from #Male#01 and

C and D are records from #Female#01.
