# Supplementary figures and images for: Density and habitat use of one of the last jaguar populations of the Brazilian Atlantic Forest: Is there still hope?
Source: Ecol Evol. 2022 Jan 15;12(1):e8487. doi: 10.1002/ece3.8487 (PMC8809435; doi:10.1002/ece3.8487)

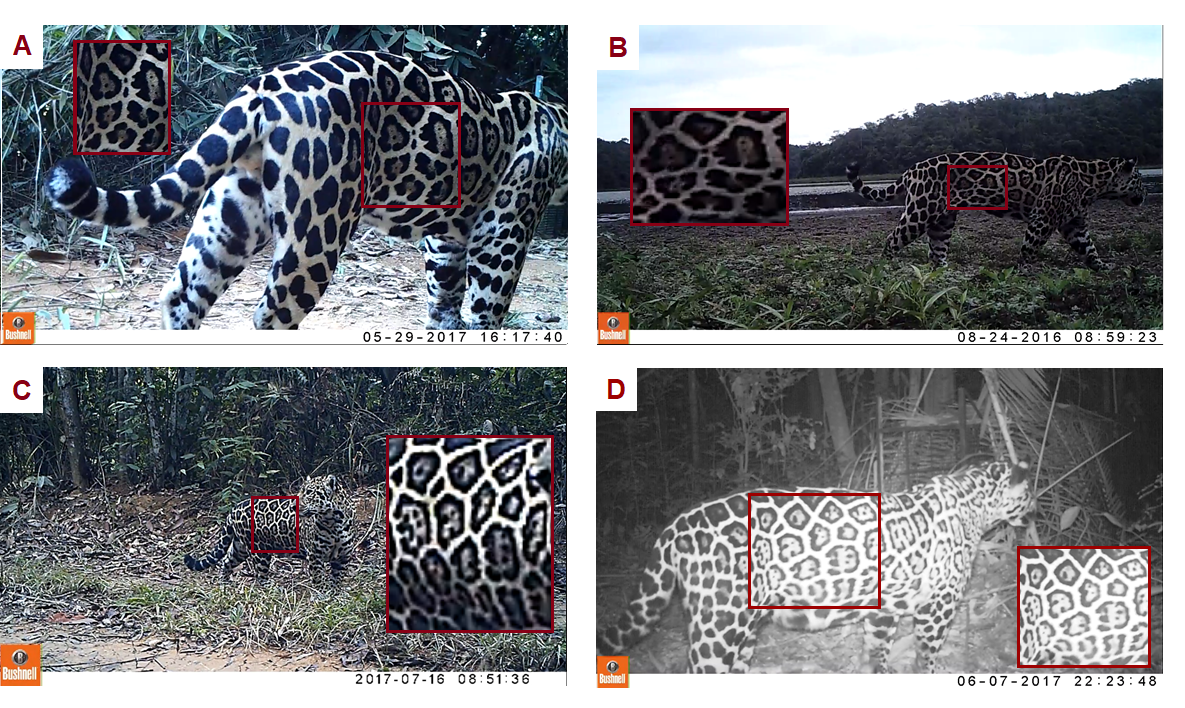

Supplement: Supplementary file 2 — Fig S1 [file ECE3-12-e8487-s001.png]
